# Transparent Conductive Nanofiber Paper for Foldable Solar Cells

**DOI:** 10.1038/srep17254

**Published:** 2015-11-26

**Authors:** Masaya Nogi, Makoto Karakawa, Natsuki Komoda, Hitomi Yagyu, Thi Thi Nge

**Affiliations:** 1 The Institute of Scientific and Industrial Research, Osaka University, 8-1 Mihogaoka, Ibaraki, Osaka 567-0047, Japan

## Abstract

Optically transparent nanofiber paper containing silver nanowires showed high electrical conductivity and maintained the high transparency, and low weight of the original transparent nanofiber paper. We demonstrated some procedures of optically transparent and electrically conductive cellulose nanofiber paper for lightweight and portable electronic devices. The nanofiber paper enhanced high conductivity without any post treatments such as heating or mechanical pressing, when cellulose nanofiber dispersions were dropped on a silver nanowire thin layer. The transparent conductive nanofiber paper showed high electrical durability in repeated folding tests, due to dual advantages of the hydrophilic affinity between cellulose and silver nanowires, and the entanglement between cellulose nanofibers and silver nanowires. Their optical transparency and electrical conductivity were as high as those of ITO glass. Therefore, using this conductive transparent paper, organic solar cells were produced that achieved a power conversion of 3.2%, which was as high as that of ITO-based solar cells.

Small, lightweight, portable electronic devices such as smart tablets and wearable devices have become widespread in everyday life. Large-area power sources such as solar cells and rechargeable batteries will be required for a long-time operation, and there is significant demand for large displays. Thus, foldable device components are a key technology for the development of future portable devices that could be folded and placed in a pocket, and then opened out at the time of use, like a newspaper or magazine.

Many electronic devices are manufactured on transparent, conductive substrates; such devices include displays and solar cells. The most commonly used transparent conductive substrates are doped metallic oxide glass, and indium tin oxide (ITO) glass is used most frequently. However, present electronic devices are heavy, and cannot be folded, because of the heavy and brittle nature of ITO glasses. To overcome these disadvantages, transparent conductive plastic substrates have been developed using carbon nanotubes, silver nanowires, or graphene instead of doped metallic oxides^1^,^2^,^3^,^4^. These transparent, conductive plastics have transparency and conductivity values as high as those of ITO glasses, but they do not have the high foldability that will be a requirement for future portable devices. Moreover, simple processes (not photolithography or laser etching) will also be required to fabricate transparent and conductive patterns on transparent substrates.

In 2009, we produced optically transparent nanofiber paper using 15-nm-wide cellulose nanofibers, which were nanofabricated from wood^5^. As a result of their excellent characteristics, which include high thermal and chemical resistances, this nanofiber paper has been successfully applied as device components in conductive electrodes^6^,^7^,^8^,^9^, antennas^10^,^11^, organic light-emitting diodes (LEDs)^12^, solar cells^13^, touch screens^14^, nonvolatile memory^15^, and transistors^16^,^17^. Here, we report the processes used to fabricate optically transparent, electrically conductive nanofiber paper using 15 nm-wide cellulose nanofibers and 50 nm-wide silver nanowires. The nanofiber paper showed electrical conductivity as high as that of ITO glass, and maintained its high conductivity after being folded many times. Finally, we demonstrated highly portable paper solar cells produced using the transparent conductive nanofiber paper; these solar cells could be folded and carried in pockets and bags.

## Experimental

### Cellulose nanofibers and silver nanowires

15-nm-wide cellulose nanofibers were mechanically nanofibrillated from wood pulps of Sitka spruce (*Picea sitchensis*), using a high-pressure water jet system (Star Burst, HJP-25005E, Sugino Machine Co., Ltd.)^5^,^18^. 0.7 wt.% cellulose nanofiber/water dispersions were used as starting materials for the optically transparent nanofiber sheets. Silver nanowires, 50–100 nm in diameter and 5–10 μm in length, were synthesized via the reduction of silver nitrate in the presence of poly(vinylpyrrolidone) (PVP) in ethylene glycol^19^. The silver nanowires were dispersed in water or ethanol to form printable inks.

### Optically transparent and electrically conductive nanofiber paper

**Figure 1:**
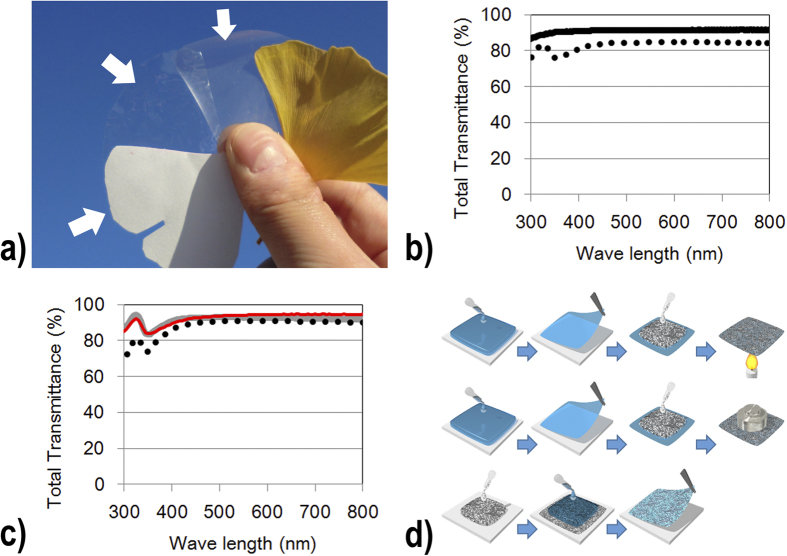
(**a**) Traditional white paper (left), transparent nanofiber paper (center), and transparent conductive nanofiber paper (right). (**b**) Optical transmittance of transparent nanofiber paper (solid line), and transparent conductive nanofiber paper (dotted line). (**c**) Optical transmittance of silver nanowire layers fabricated on the transparent nanofiber paper using a heating method (dotted line), pressing (gray line), and dropping (red line). (**d**)Transparent conductive nanofiber paper produced using the heating method (upper), pressing method (middle), and dropping method (lower). The cellulose nanofiber dispersion is shown as blue, and the silver nanowire suspension is shown as black.

Optically transparent and electrically conductive nanofiber paper was fabricated using the three methods of heating, mechanical pressing, and deposition via dropping (Fig. 1d). In the heating and mechanical pressing methods, the optically transparent nanofiber papers were prepared before the deposition of the silver nanowires. The fabrication of the transparent nanofiber paper was achieved as follows: The nanofiber dispersions were dropped onto a silicon wafer and were then oven-dried at 50 °C for 1 day. After drying, an optically transparent nanofiber paper with a thickness of 15–20 μm was obtained^18^.

**Figure 2:**
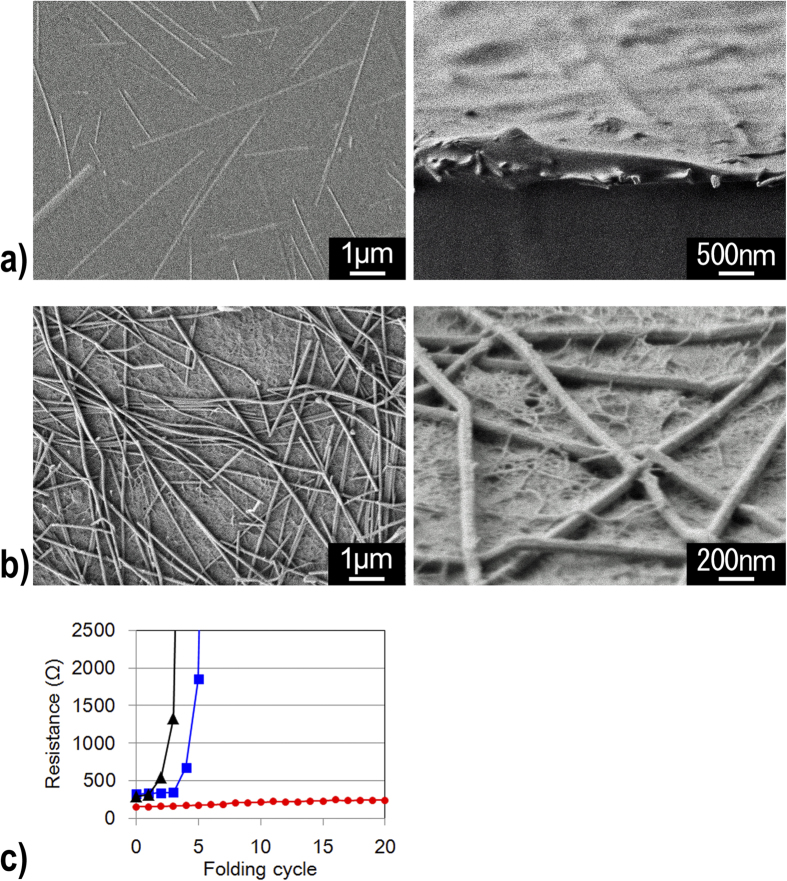
(**a**) Silver nanowires were buried in the PVA substrate (left: top view, right: side view). (**b**) Silver nanowires were deposited on the transparent nanofiber paper, and were entangled with the cellulose nanofibers (left: top view, right: side view). (**c**) Electrical resistance of transparent silver nanowires on a PET film (black), a PVA film (blue), and transparent nanofiber paper (red), as a function of the number of folding cycles, performed in zero-span roll-tests.

(1) Heating method (Fig. 1d): A 0.3 wt.% silver nanowire/ethanol suspension was bar-coated onto the transparent nanofiber paper, and then air-dried for 3–5 min. The air-dried silver nanowires on the nanofiber papers were heated at 150 °C for 30 min in air. (2) Mechanical pressing method (Fig. 1d): A 0.3 wt.% silver nanowire/ethanol suspension was bar-coated onto the transparent nanofiber papers, and was then air-dried for 3–5 min. The air-dried silver nanowire networks on the nanofiber papers were mechanically pressed at 2 MPa and 20 °C for 20 s. Using a polyethylene terephthalate (PET) film as a transparent substrate, as shown in Fig. 2c, air-dried silver nanowire networks were pressed at 10 MPa and 20 °C for 20 s. (3) Dropping method (Fig. 1d): A 0.3 wt.% silver nanowire/water suspension was cast on a silicon wafer, and then air-dried. A 0.7 wt.% cellulose nanofiber/water dispersion was cast over the dried silver nanowire layer on a silicon wafer, and then air-dried at 50 °C for 12–24 hours. After drying, the nanofiber paper was removed from the silicon wafer. The obtained optically transparent nanofiber paper with a silver nanowire layer was 15–20 μm thick.

### Folding tests on the transparent conductive films with silver nanowires

Silver nanowire patterns on nanofiber paper (produced using a dropping method), silver nanowire patterns on PVA films (produced using a dropping method), and silver nanowire patterns on PET substrates (produced using a heating method) were subjected to the folding tests. The pattern was 3 mm wide and 50 mm long, and the thickness of all of the substrates was 50 μm. The samples were folded across the center, to −180° (silver patterns inside), and were then repeatedly passed through rollers with a gap of approximately 100 μm. The electrical resistance was measured using a two-point probe method (34410A, Agilent).

### Organic solar cells

Organic solar cells were fabricated on nanofiber papers with silver nanowires, and on conventional ITO glass. Optically transparent and electrically conductive nanofiber papers were fabricated with silver nanowires using a pressing method. Before deposition of active layer and transparent anode, conductive nanofiber paper was laminated on glass substrate using a double-sided tape. The transparent anodes were coated with a layer of poly(3,4-ethylenedioxythiophene) doped with polystyrene sulfonate (PEDOT: PSS) using spin-coating applied at 500 r/min for 5 s, and at 3000 r/min for 60 s. A blend of poly(3-hexylthiophene): [6,6]-phenyl C61 butyric acid methyl ester (P3HT/PCBM) in chlorobenzene solution was spin-coated (at 1500 r/min, for 60 s) on top of the PEDOT: PSS coating on the transparent anode, and a 60 nm Al cathode was vacuum evaporated at 10–5 Torr. The active device area was 3 × 3 mm^2^.

### Characterization

The total light transmittance spectrum of the nanofiber paper was measured at wavelengths from 200 to 800 nm, using a UV-visible spectrometer with an integrating sphere (U-3900, Hitachi High-Tech. Corp.). The haze was measured using a haze meter (HZ-V3, Suga Test Instruments Co., Ltd.). The sheet resistance was measured using the four-point probe method (MCP-T610 Loresta type, Mitsubishi Chemical Analytech Co., Ltd.).

## Results and Discussion

Traditional paper—which is typically fabricated using 15–50-μm-wide cellulose pulp fibers—is white and opaque because the cavities between the fibers produce light scattering (Fig. 1a). In contrast, the nanofiber paper produced using 15-nm-wide cellulose fibers exhibited high optical transparency (Fig. 1a), because the densely packed cellulose nanofibers did not produce light scattering either inside the paper or at its surfaces^5^. The nanofiber paper showed a high total transmittance of 91.4% at a wavelength of 600 nm (Fig. 1b), a value as high as theoretically predicted values^18^. However, the optically transparent nanofiber paper does not itself have any electrical conductivity; therefore, high electrical conductivity was achieved here in the optically transparent nanofiber paper via the deposition of a silver nanowire thin film (Fig. 1c).

Silver nanowires synthesized using the polyol process have diameters in the range 50–100 nm, and are surrounded by the insulating polymer PVP^1^,^19^. When these silver nanowires are deposited on transparent substrates, they maintain the high optical transparency of the substrate. However, as-deposited networks of these silver nanowires do not display high conductivity, because the surface PVP prevents electrical contact between the silver nanowires. To increase their conductivity, silver nanowires on transparent substrates should be heated to above 150 °C^1^,^19^. This temperature is too high for commonly used plastic substrates, but such high temperatures do not damage the transparent nanofiber paper. After the silver nanowires were deposited on the nanofiber paper and heated at 150 °C for 30 min (Fig. 1d), the thin layer of silver nanowires exhibited a low sheet resistance of 39 Ω/square, and a high optical transmittance of 91.0% at 600 nm (Fig. 1c). Mechanical pressing at room temperature can also be used to enhance the conductivity of silver nanowire networks^19^. Transparent nanofiber paper is a high-strength material, because it consists of nanofibers that have high mechanical strengths of 1.6–3 GPa^20^ . The silver-nanowire-coated nanofiber paper was subjected to mechanical pressing to further enhance the conductivity of the silver nanowire networks (Fig. 1d). In our previous study, silver nanowires deposited on PET films or glass substrates were exposed to pressures greater than 10 MPa to obtain low sheet resistances of less than 50 Ω/square^19^. Here, exposure to just 2 MPa yielded a low sheet resistance of 43 Ω/square in the thin layer of silver nanowires, with a high optical transmittance of 92.8% (Fig. 1c). Because of the high thermal and mechanical durability of the cellulose nanofiber paper, these transparent and conductive nanofiber papers exhibited sheet resistance and optical transmittance values as good as those of ITO glass.

These two types of transparent and conductive nanofiber paper were fabricated using the following steps: making the transparent nanofiber paper; depositing the silver nanowire suspensions; and performing post treatments consisting of heating or mechanical pressing (Fig. 1d). As an alternative to these time- and labor-intensive processes, we also developed a simple procedure that did not require any post treatment (Fig. 1d). First, silver nanowire suspensions were deposited on the silicon wafer drying plate. Next, cellulose nanofiber dispersions were dropped on the dried silver nanowire layer. These samples were dried, and the transparent nanofiber paper was obtained by peeling the sample off the plate. As mentioned above, the as-deposited silver nanowire layer did not have a high conductivity. When a cellulose nanofiber/water dispersion (99.3 wt% water, 0.7 wt% nanofibers) was dried, the final volume of the dispersion was less than 1% of the original volume (after the water had evaporated). During the drying process, the drop dimensions decreased only in thickness; the spreading area was maintained. This anisotropic shrinkage had a mechanical pressing effect, thus increasing the number of electrical contacts between the silver nanowires, as Zhu *et al*. has also suggested^21^. As a result, the obtained silver nanowire layer displayed a maximum transmittance of 94.4% at a wavelength of 600 nm (Fig. 1c), and a minimum sheet resistance of 17 Ω/square. As a result, the transparent nanofiber paper had an electrical conductivity that was as high as that of ITO glass, without any loss in the high optical transparency (Fig. 1b).

Polymer solutions could also be dropped on the silver nanowire networks^22^,^23^. When a PVA solution was dropped on the silver nanowires, the obtained film showed high optical transparency and high electrical conductivity. However, because the PVA solution penetrated between the silver nanowires (Fig. 2a), the transparent and conductive PVA film had a high sheet resistance of 297 Ω/square at 95% transmittance. In contrast, when cellulose nanofiber dispersions were cast on the silver nanowires, the silver nanowires remained on the surface of the cellulose nanofiber networks (Fig. 2b). Because the cellulose nanofibers were more than several dozen micrometers in length, they could not penetrate between the silver nanowires, which defined cavities with dimensions smaller than a few micrometers. As a result, the transparent and conductive cellulose nanofiber paper exhibited a low sheet resistance of 148 Ω/square, less than half of the sheet resistance of the transparent and conductive PVA film (297 Ω/square) at a transmittance of 95%.

The dropped transparent and conductive nanofiber paper exhibited high electrical durability in repeated folding tests (Fig. 2c). Because the silver nanowires were surrounded by hydrophilic PVP, there was a low adhesion strength between the silver nanowires and the hydrophobic polymer substrates. When a hydrophobic PET film with silver nanowire layers was folded four times, the conductivity was lost, because of the removal of the silver nanowire layer from the PET film. However, the silver nanowire layers on the hydrophilic PVA film maintained their conductivity after five folding cycles, as a result of the good affinity between the PVA substrate and the hydrophilic PVP. Notably, the silver nanowire layers on the transparent nanofiber paper maintained their high conductivity even after twenty folding cycles. The high electrical durability of the nanofiber paper did not result only from the high affinity between the PVP on the silver nanowires and the cellulosic nanofiber paper. Careful observations of the conductive nanofiber paper showed that the silver nanowires were entangled in the cellulose nanofibers (Fig. 2b). Therefore, the high adhesion strength against folding was enhanced by the dual advantages of the hydrophilic affinity between the PVP and the cellulose, and the entanglement between the silver nanowires and the cellulose nanofibers.

**Figure 3:**
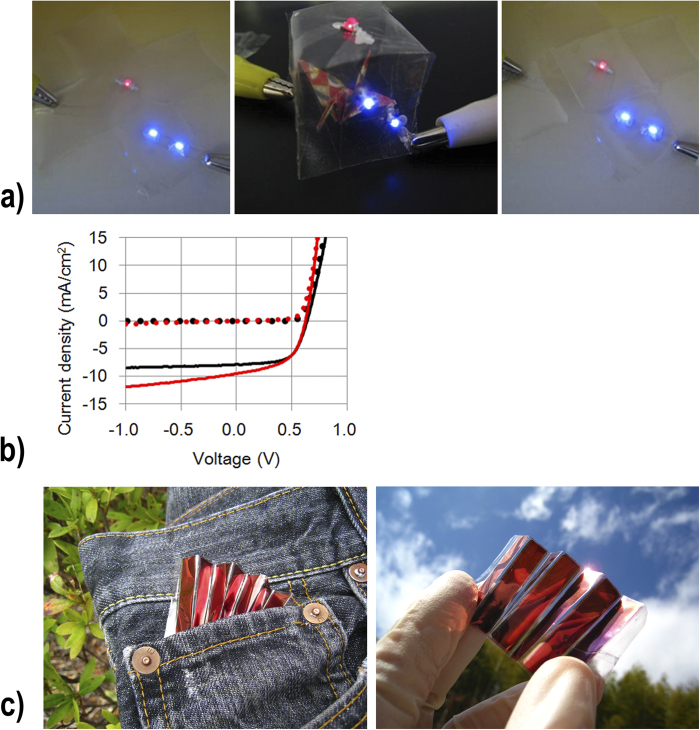
(**a**) Operation of light-emitting diodes using foldable transparent and conductive silver nanowire patterns on transparent nanofiber paper (left: before folding, center: under folding, right: after recovery to the original shape). (**b**) Current–voltage characteristics of the organic solar cells (P3HT/PCBM) in the dark (broken lines), and under 100 mW/cm^2^ of AM 1.5 G illumination (solid lines); Red plot: transparent conductive nanofiber paper-based solar cells; black plot: indium tin oxide glass-based solar cells. (**c**) Portable paper solar cells based on foldable and lightweight transparent conductive nanofiber paper.

This procedure produced not only high electrical durability, but also transparent conductive patterns. When the silver nanowire inks were printed on a drying plate, and then the peeling off of the nanofiber paper (Fig. 1d), transparent and conductive patterns were fabricated on the nanofiber paper without the use of any etching processes. The transparent silver nanowire patterns on the nanofiber paper could be used to illuminate LED lights, as a result of their high electrical conductivity (Fig. 3a). The LED lights could still be illuminated under folding, and after recovery to the original flat form, because of the high foldability of the devices (Fig. 3a).

The transparent nanofiber paper was used to fabricate paper solar cells, via the printing of organic solar cell components on the transparent conductive nanofiber paper. We fabricated organic solar cells based on ITO glass with an active layer of P3HT/PCBM, their short current density was 7.89 mA/cm^2^, and their power conversion efficiency was 3.1% (Fig. 3b). In the paper solar cells, we used the transparent nanofiber paper instead of glass, and silver nanowires instead of ITO electrodes. In previous studies of paper solar cells, the measured power conversion efficiency was less than one-tenth, or half, than that of ITO-based solar cells, even when the same active solar layer was used^13^,^24^. Our transparent conductive nanofiber paper had optical transparency and electrical conductivity values as high as those of ITO glass, as mentioned above. In our study, the nanofiber paper consisted of native cellulose fibers, which have high chemical durability. Therefore, they maintained their high optical transparency and high electrical conductivity after coating with acid PEDOT:PSS and P3HT/PCBM chlorobenzene solutions. Moreover, the conductive nanofiber paper did not have dimensional change such as wrinkle and shrinkage during a coating process, since it was laminated on glass substrate using a double-sided tape. As a result, our paper solar cell achieved a power conversion efficiency of 3.2%, as high as that of ITO-based solar cells, and a short current density of 9.58 mA/cm^2^ (Fig. 3b). Moreover, we found that the nanofiber paper solar cells exhibited power conversion under folding, and after folding. The nanofiber solar cells could therefore supply electric power everywhere, while (and after) being carried in a pocket or bag (Fig. 3c).

## Conclusions

In conclusion, we reported optically transparent conductive paper produced using cellulose nanofibers and silver nanowires. The optical transparency and electrical conductivity of the optically transparent conductive paper were as high as those of ITO glass. Paper solar cells were fabricated using the transparent conductive paper; these paper solar cells exhibited a high power conversion efficiency of 3.2%, equal to that of ITO glass-based solar cells. Because of the high affinity and high degree of entanglement between the cellulose nanofibers and the silver nanowires, the nanofiber paper maintained its high conductivity—and the paper solar cells still generated electrical power—under folding, and after folding. Moreover, transparent conductive patterns were successfully formed on the nanofiber paper via the printing of silver nanowires. We believe that this highly transparent conductive nanofiber paper will play an important role in future portable electronics.

## Additional Information

**How to cite this article**: Nogi, M. *et al*. Transparent Conductive Nanofiber Paper for Foldable Solar Cells. *Sci. Rep*. **5**, 17254; doi: 10.1038/srep17254 (2015).
